# Viral-Porphyrin Combo: Photodynamic and Oncolytic Viral Therapy for Potent Glioblastoma Treatment

**DOI:** 10.3390/ijms252312578

**Published:** 2024-11-22

**Authors:** Alina S. Nazarenko, Alena O. Shkirdova, Ekaterina A. Orlova, Yulia K. Biryukova, Mikhail F. Vorovitch, Nadezhda M. Kolyasnikova, Aydar A. Ishmukhametov, Vladimir S. Tyurin, Ilya A. Zamilatskov

**Affiliations:** 1 Chumakov Federal Scientific Center for Research and Development of Immune-and-Biological Products of Russian Academy of Sciences (Institute of Poliomyelitis), 108819 Moscow, Russiaorlova_ea@chumakovs.su (E.A.O.); vorovich_mf@chumakovs.su (M.F.V.); kolyasnikova_nm@chumakovs.su (N.M.K.);; 2 Frumkin Institute of Physical Chemistry and Electrochemistry, Russian Academy of Sciences, 119071 Moscow, Russia; 3 Institute for Translational Medicine and Biotechnology, Sechenov University, 117418 Moscow, Russia; 4 Chair of Chemistry, The Institute of Pharmacy, Sechenov First Moscow State Medical University (Sechenov University), 119571 Moscow, Russia; joz@mail.ru

**Keywords:** vesicular stomatitis virus, targeted delivery, viral therapy, photodynamic therapy, porphyrin, tumor, glioblastoma, combination therapy

## Abstract

Combined viral and photodynamic therapy for oncological diseases has great potential to treat aggressive tumors such as glioblastomas. A conjugate of vesicular stomatitis virus (VSV) with protoporphyrin IX was prepared, and its oncolytic effects were studied and compared to the effects of the individual components. The VSV showed an oncolytic effect on glioblastoma cell lines T98G and LN229 at a virus titer of 10^5^ TCID_50_/mL. A VSV titer of 10^4^ TCID_50_/mL was sufficient for neuroblastoma cell death. A study of the effect of VSV in tumor 3D cell modeling found that VSV had a clear viral cytopathic effect on spheroids of T98G and LN229 cells. Conjugation with the porphyrin significantly reduced the viral titer, but when irradiated, lysis of cells was observed. Photodynamic treatment of T98G and LN229 cells and spheroids with protoporphyrin IX as a photosensitizer also had a cytotoxic effect on cells and, to a lesser extent, on the tumoroids, as complete cell death was not achieved for the tumoroids. The combination therapy, which involved sequential photodynamic therapy using protoporphyrin IX as a photosensitizer and treatment with VSV, was shown to significantly enhance efficacy, resulting in complete cell death of both T98G and LN229 cells and tumoroids. The combination treatment allowed for the use of a lower viral titer (10^3^–10^4^ TCID_50_/mL) and a lower porphyrin concentration (0.5 μg/mL) to achieve a significant cytotoxic effect. As a result, the implementation of this combination therapy would likely lead to fewer side effects from the treatment. This study clearly demonstrated the excellent perspectives of combination therapy for the treatment of highly aggressive tumors such as glioblastomas.

## 1. Introduction

Glioblastomas (GBs) are among the most aggressive types of tumors, as they have a high rate of proliferation, the ability to infiltrate other tissues, and resistance to conventional treatments. This results in a poor survival prognosis [1,2]. Treatment of glioblastomas presents a significant challenge, highlighting the critical need for research into novel therapeutic strategies.

Oncolytic virotherapy is a promising cancer treatment that uses viruses to selectively target and destroy tumor cells while stimulating the immune system [3,4]. One of the key advantages of this approach is its independence from detailed knowledge of tumor-specific antigens, allowing for its applicability across a wide range of cancer types.

Oncolytic viruses (OVs) can identify, infect, and destroy various transformed cells. They can also stimulate antitumor immunity. Due to their natural neurotropism, alpha- and flaviviruses [5] can be utilized for glioblastoma treatment through the use of attenuated viral strains. These viruses are attractive as oncolytic agents due to their ability to rapidly and extensively replicate RNA in the cytoplasm, providing extreme levels of transgene expression that can be advantageous in cancer therapy. Viruses belonging to the Rhabdoviridae family have a single-stranded, negative-sense RNA genome. Some of these viruses can cross the blood–brain barrier, making them useful for treating brain tumors. Poliovirus, a member of the Picornavirus family with a single-stranded, positive-sense RNA genome, shows promise as an oncolytic virus. Due to its ability to cross the blood–brain barrier, genetically engineered variants of the virus are actively being developed for the treatment of brain tumors [6,7,8]. Vesicular stomatitis virus (VSV) is an oncolytic virus that demonstrates multiple mechanisms of tumor cell destruction. These mechanisms include direct cell lysis, hypoxia caused by the shutdown of the tumor’s blood vessels, and the release of inflammatory cytokines [9].

Current research efforts are particularly concentrated on optimizing the interactions of these viruses with the tumor microenvironment (TME). This includes transforming the typically immunosuppressive environment into one that promotes inflammation, thereby enhancing the effectiveness of other therapies such as immune checkpoint inhibitors and cell-based treatments. Additionally, improving virus delivery systems and minimizing side effects are crucial for increasing success in clinical applications [10,11].

Oncolytic viruses are a promising approach for glioblastoma treatment due to their ability to adapt to the brain environment, selectively target fast-growing tumor cells, and transform the immunosuppressive microenvironment into an immune-responsive one, stimulating an antitumor immune response [12,13,14]. Current clinical trials are investigating these viruses as part of novel therapies, combining direct cytotoxic effects with immune activation to enhance tumor clearance and improve patient survival, suggesting their potential role in future standard treatments for glioblastomas [15].

Despite the potential, the long-term success of viral immunotherapy in solid tumors has been limited, largely due to the immunosuppressive nature of the tumor microenvironment and insufficient immune cell infiltration. The TME consists of various components, including tumor cells, tumor-associated fibroblasts, endothelial cells, mesenchymal cells, myeloid-derived suppressor cells, and tumor-infiltrating immune cells such as T and B cells, dendritic cells, natural killer cells, and macrophages [16]. These immune cells, including exhausted cytotoxic T lymphocytes, helper T cells, and natural killer cells, alongside regulatory T cells, tolerogenic dendritic cells, myeloid-derived suppressor cells, and M2 macrophages contribute to the immunosuppressive environment by releasing inhibitory cytokines such as interleukin-10, transforming growth factor-beta, interleukin-35, and interleukin-27 [17].

Combining oncolytic viruses with traditional and novel therapies has shown significant potential in treating glioblastoma. This approach enhances the antitumor effect, with studies demonstrating that combining viral therapy with standard treatments prolongs patient survival. OVs used alongside chemo- and radiotherapy create a synergistic effect, increasing tumor cell apoptosis, while immune checkpoint inhibitors and CAR T-cell therapy further improve the immune response and therapeutic outcome [18].

Photodynamic therapy (PDT) is a promising, minimally invasive treatment option for oncological diseases [19,20], including glioblastomas [21,22]. It has been shown to be effective in reducing the risk of local recurrence after tumor resection [23]. The introduction of a photosensitizer, followed by direct irradiation of the tumor mass using stereotactic insertion of a light source (an optical fiber connected to a laser diode), without craniotomy, leads to a minimally invasive treatment known as interstitial photodynamic therapy (iPDT) [24]. A recent study has demonstrated the safety and effectiveness of iPDT in brain tumors, showing promising results for glioblastoma treatment [25]. However, one of the challenges of PDT is increasing the selectivity of photosensitizer accumulation in tumor cells and tissues [26,27,28]. This is crucial for ensuring the localization of treatment effects and minimizing side effects. It is known that porphyrins have the ability to selectively accumulate in tumor tissues. This property, along with their optimal photophysical characteristics for PDT, has led to their widespread use as photosensitizers [29]. Several porphyrin photosensitizers have been approved for use in a number of countries to treat malignant tumors, including breast, bladder, and esophageal cancers [30].

In recent years, the combined use of photodynamic therapy and oncolytic viruses has garnered increasing attention in oncology research due to its potential to enhance therapeutic outcomes. PDT operates by activating photosensitizers that, under light exposure, generate reactive oxygen species, leading to tumor cell damage and destruction. Additionally, PDT can affect the tumor’s vascular system by disrupting its blood supply and increasing permeability, which facilitates the entry of other therapeutic agents, such as oncolytic viruses, thus creating opportunities for a synergistic approach [31,32].

Research indicates that the combination of PDT and OVs improves viral penetration into tumor tissue by altering the tumor microenvironment, particularly its vascular structure, enhancing viral replication and antitumor effects [33]. Moreover, the combination of PDT and OVs promotes immunogenic cell death, which is crucial for triggering a more active immune response against the tumor. The second-generation chlorin-based photosensitizer, 2-(1-hexyloxyethyl)-2-devinyl pyropheophorbide-a (HPPH), has shown improved photophysical and pharmacokinetic properties compared to Photofrin in clinical trials [32].

Preclinical studies from 2004 demonstrated that HPPH-PDT can trigger diverse vascular, cellular, and inflammatory responses, with tumor control depending on the treatment protocol. Combining HPPH-PDT with a genetically modified vaccinia virus (VV) has shown potential for enhancing treatment efficacy. Tested on NXS2 neuroblastoma models and human FaDu xenografts, the combination therapy demonstrated superior efficacy in controlling tumor growth compared to either treatment administered individually. Notably, one dosage combination led to a reduction in tumor volume within six weeks. PDT’s ability to disrupt tumor vasculature increased the viral load in tumors, illustrating the synergistic effect of the two therapies [34].

Thus, the combined use of photodynamic therapy and oncolytic viruses is a promising direction for treating aggressive tumors like glioblastomas due to its multi-mechanistic action, including direct tumor cell destruction, modification of the tumor microenvironment, and immune system activation [35].

## 2. Results

### 2.1. Oncolytic Properties of Vesicular Stomatitis Virus on Human Glioblastoma Cell Lines GL6, LN229, T98G, Human Neuroblastoma SH5y5y and Kelly, as well as on Mouse Glioma GL261

**Figure 1:**
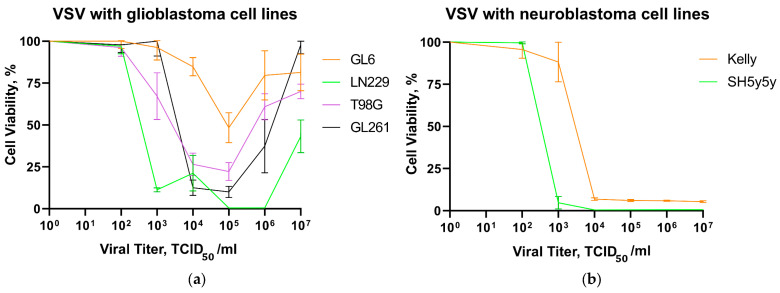
The sensitivity of human and mouse cell lines to VSV, assessed by measuring cell viability using the MTT assay after exposure to various titers of VSV for 3 days: (**a**) glioblastoma cell lines; (**b**) neuroblastoma cell lines.

Initially, we evaluated the oncolytic properties of VSV on human glioblastoma cell lines GL6, LN229, T98G, and human neuroblastoma SH5y5y and Kelly, as well as on the mouse glioma cell line GL261, using the MTT test. The cells were infected with VSV at titers ranging from 10^2^ to 10^7^ TCID_50_/mL and incubated for 3 days. We found that at a VSV titer of 10^5^ TCID_50_/mL, the LN229 cells died completely, while the survival rates of T98G, GL261, and GL6 were 22 ± 5%, 10 ± 3%, and 48 ± 9%, respectively (Figure 1a). Further increase in virus titer led to a decrease in the oncolytic activity of VSV. Complete cell death was also observed in neuroblastomas at a titer of 10^4^ TCID_50_/mL (Figure 1b).

### 2.2. The Cytotoxic Effect of Protoporphyrin IX on Human Glioblastoma Cell Lines

**Figure 2:**
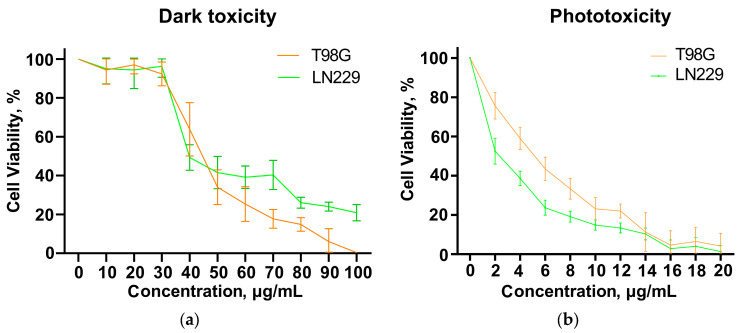
The sensitivity of human and mouse glioblastoma cell lines to protoporphyrin IX, assessed by measuring cell viability after incubation of cells in PBS buffer with porphyrin solution in DMSO, using the MTT assay: (**a**) dark toxicity (incubation in the dark for an hour); (**b**) phototoxicity (after one hour of incubation, the cells were irradiated with light at a wavelength of 450 nm).

Evaluation of the cytotoxicity and phototoxicity of protoporphyrin IX was performed on T98G and LN229 cells using the MTT test. The results showed that the porphyrin exhibited dark toxicity with an IC50 of about 48 ± 7 μg/mL for T98G cultures, while the IC50 for LN229 was 40 ± 6 μg/mL (Figure 2a). Treatment with protoporphyrin IX followed by light irradiation had a cytotoxic effect on the studied cell lines in a dose-dependent manner, with an IC50 observed at a concentration of 5 ± 1 μg/mL for T98G and about 3 ± 1 μg/mL for LN229 (Figure 2b).

### 2.3. Conjugation of Dyes with VSV

**Scheme 1:**
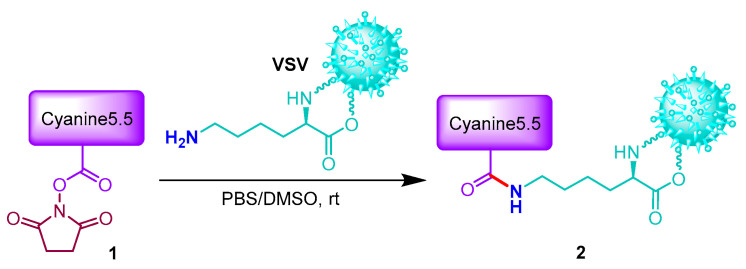
Synthesis of the conjugate of VSV with Cyanine5.5 (**2**).

**Scheme 2:**
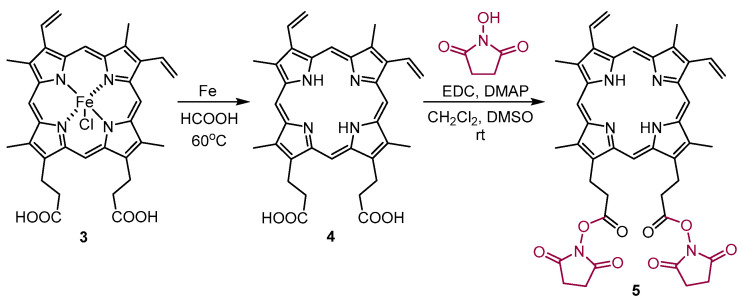
Synthesis of the N-succinimide ester of protoporphyrin IX (**5**).

**Scheme 3:**
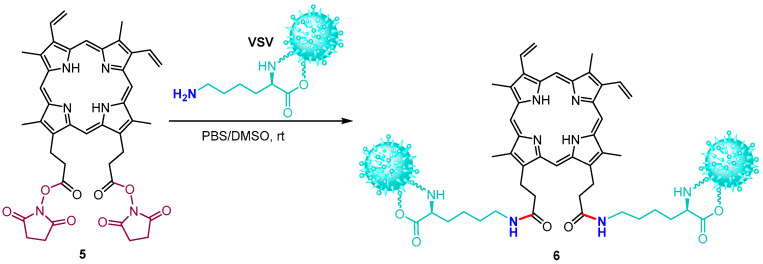
Synthesis of the conjugate of protoporphyrin IX with VSV (**6**).

The ability of VSV to interact with small molecules without losing its infectivity was tested using a model reaction with the fluorescent dye Cyanine5.5. Purified and concentrated VSV at a titer of 10^5^ TCID_50_/mL was mixed with a solution of the activated derivative of Cyanine5.5 in the form of its N-succinimide ester (**1**), followed by purification of the resulting conjugate (**2**) from unreacted dye (Scheme 1). In the next step, the conjugate of VSV with protoporphyrin IX was obtained using a similar method as for the conjugation with Cyanine5.5. For this purpose, the activated derivative of protoporphyrin IX was obtained using the following procedure: Protoporphyrin IX (**4**) was prepared from hemin (**3**) followed by the synthesis of the N-succinimide ester (**5**) (Scheme 2). Then, VSV at a titer of 10^5^ TCID_50_/mL was mixed with the activated porphyrin derivative **5**, followed by purification of the resulting conjugate (**6**) from unreacted porphyrin (Scheme 3).

**Figure 3:**
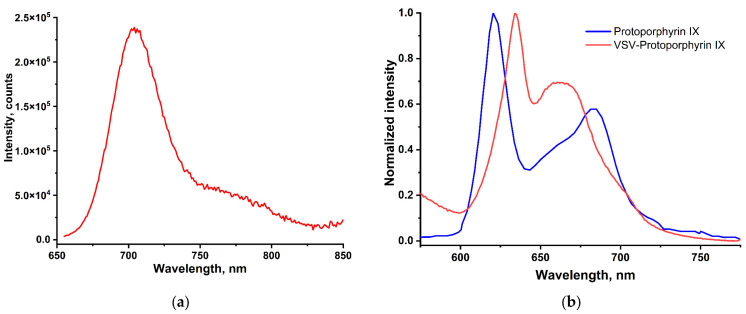
Fluorescence spectra of (**a**) VSV–Cyanine5.5 conjugate (excitation at 635 nm); (**b**) VSV–protoporphyrin IX conjugate (excitation at 405 nm) (red line) and protoporphyrin IX (blue line). Measured in PBS buffer.

The fluorescence spectrum of the VSV–Cyanine5.5 conjugate (**2**) in PBS buffer fully corresponded to that of the pure Cyanine5.5 dye, with a maximum at 704 nm and a shoulder at 752 nm (Figure 3a). The fluorescence spectrum of the conjugate of VSV with protoporphyrin IX (**6**) exhibited a bathochromic shift of the maximum by 14 nm (Figure 3b).

**Figure 4:**
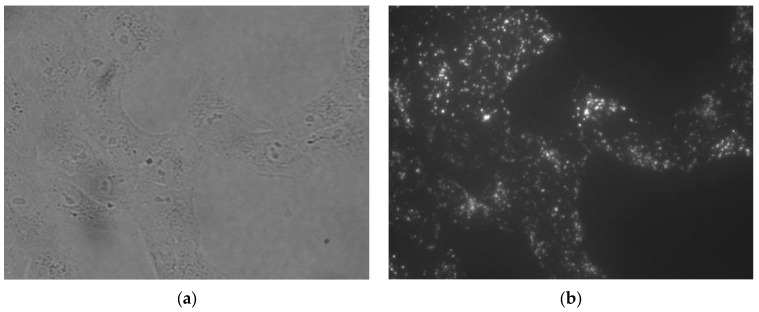
VSV conjugated with Cyanine5.5 on the Vero cell line: (**a**) at full light spectrum; (**b**) channel Cy5. The images were captured at 40× magnification using the Celena X imaging system.

To evaluate the infectivity of the resulting conjugates, we performed an infection experiment using the Vero cell line at a passage density of approximately 40%. After that, we assessed the fluorescent signal produced by the conjugate. We observed the adsorption of viral particles on the cell surface and the movement of capsids along the cytoskeleton (Figure 4) for the VSV–Cyanine5.5 conjugate. Two days later, we also observed the cytopathic effect of VSV, which is characteristic of viral infection. However, it should be noted that the titer decreased significantly in comparison to the original virus by five orders of magnitude.

**Figure 5:**
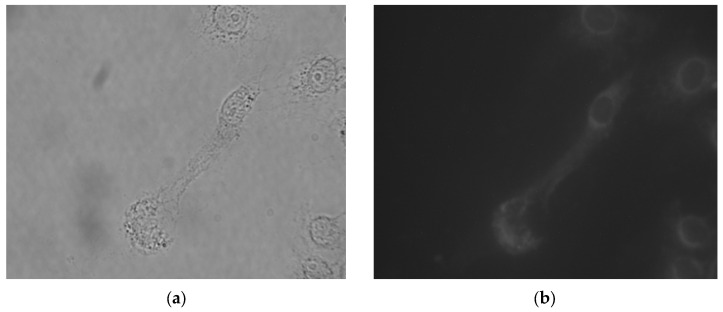
VSV conjugated with protoporphyrin IX (**4**) on the Vero cell line: (**a**) at full light spectrum; (**b**) at red light spectrum. The images were captured at 40× magnification using the Celena X imaging system.

The VSV–porphyrin conjugate (**4**) remained infectious, as confirmed by a fluorescent signal along the Vero cell boundaries (Figure 5) and a viral cytopathic effect that we observed on the second day after infection; however, titration of the conjugate showed an 8-fold decrease in virus titer.

**Figure 6:**
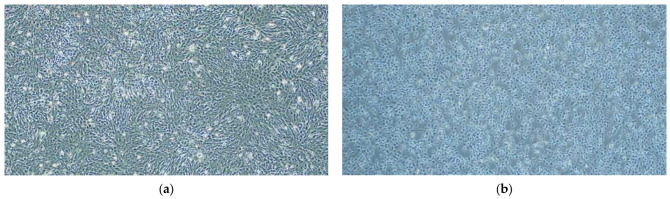
(**a**) Uninfected T98G culture; (**b**) VSV–protoporphyrin IX conjugate (**4**) on the T98G cell line after light exposure. Light microscopy; magnification: 4×.

The phototoxic properties of the resulting VSV–porphyrin conjugate (**4**) were tested on the T98G cell line. First, the monolayer of T98G cells was infected with the conjugate, followed by exposure to light at a wavelength of 450–480 nanometers. The next day, we observed cell lysis compared to the control group (Figure 6). However, complete cell death was not detected.

### 2.4. The Combination of Photodynamic and VSV Treatments Enhances the Lysis of Human Glioblastoma Cell Lines

**Figure 7:**
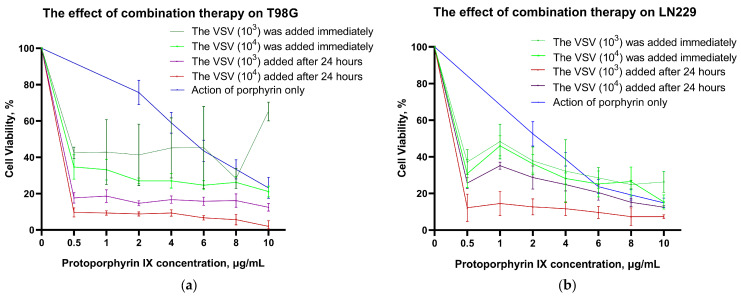
The survival rate after treatment of the cell lines: (**a**) T98G; (**b**) LN229. The cells were initially incubated with various concentrations of protoporphyrin IX for one hour, followed by irradiation at a wavelength of 450 nm. Then, the cultures were treated with VSV at titers of 10^3^ and 10^4^ TCID_50_/mL immediately after irradiation or one day later. Cell viability was determined using the MTT assay.

The combined lytic effect of protoporphyrin-IX-sensitized photodynamic and VSV treatments on T98G and LN229 cell lines was studied using the MTT assay. The cells were initially incubated with various concentrations of protoporphyrin IX for one hour, followed by irradiation at a wavelength of 450 nm. Then, the cultures were treated with VSV at titers of 10^3^ and 10^4^ TCID_50_/mL immediately after irradiation or one day later. The MTT assay was performed three days after the viral treatment. First, we analyzed T98G cell survival after viral treatment immediately following photodynamic treatment. Average T98G cell survival at a VSV titer of 10^4^ TCID_50_/mL varied between 21% and 35%, depending on the concentration of protoporphyrin IX (Figure 7a). In cultures treated with the virus alone, the average cell survival was 27 ± 5%. When the virus was added at a titer of 10^3^ TCID_50_/mL, the average cellular survival rate was approximately 50 ± 12% for both viral monotherapy and combination therapy. Treatment of T98G cells with the porphyrin alone resulted in a 23–76 ± 6% survival rate. Alternatively, viral treatment was applied one day after the porphyrin-sensitized photodynamic treatment. At a titer of 10^4^ TCID_50_/mL, the average cell survival was 2–10 ± 2%, while at a titer of 10^3^ TCID_50_/mL, it was 13–18 ± 3%. The survival rates varied depending on the dose of porphyrin used.

The same treatment of LN229 cells showed no effect of combination therapy when the virus was added immediately after photodynamic treatment. However, there was a slight increase in lytic activity when the virus was administered one day later (Figure 7b). When the cells were treated with the virus alone at a concentration of 10^4^ TCID_50_/mL, the average cellular survival rate on day 3 was approximately 20 ± 11%. After combination therapy, the survival rate ranged from 35% to 13 ± 3%, depending on the dose of protoporphyrin IX (Figure 7b). Using the virus as monotherapy with a titer of 10^3^ TCID_50_/mL had the same lytic effect of about 17 ± 4%. However, with combination therapy, we observed an increase in the effect: cellular survival ranged from 15% to 7 ± 4%.

### 2.5. Effect of VSV on 3D Spheroids

**Figure 8:**
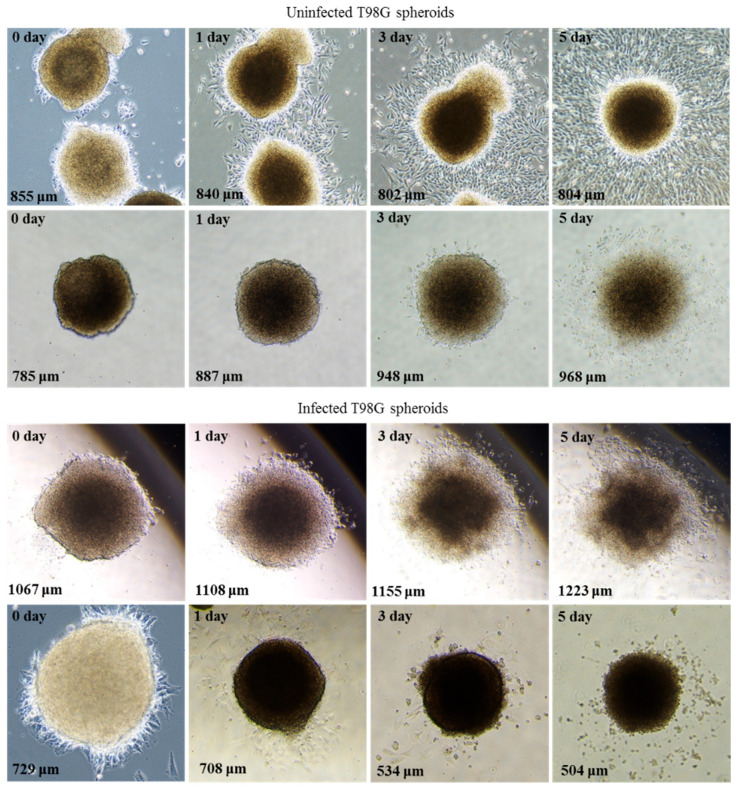
The effect of VSV on T98G spheroids. Spheroids were infected with VSV at a titer of 10^5^ TCID_50_/mL with the addition of VSV culture medium and compared with control uninfected spheroids. Light microscopy photographs of the spheroids were taken daily and presented at a magnification of 4×; the measured size of the spheroid is indicated in the lower left corner.

**Figure 9:**
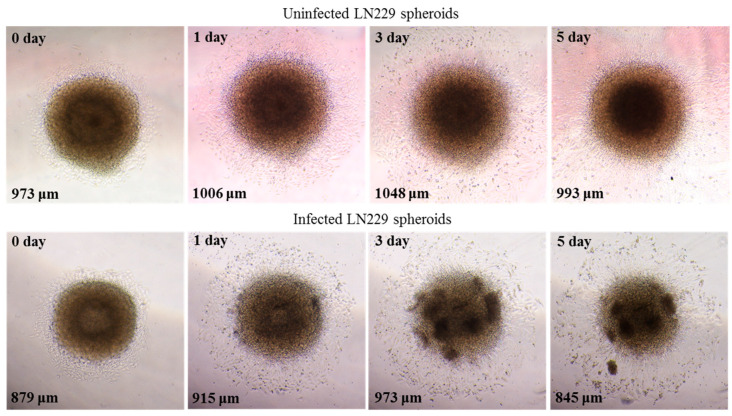
The effect of VSV on LN229 spheroids. Spheroids were infected with VSV at a titer of 10^5^ TCID_50_/mL with the addition of VSV culture medium and compared with control uninfected spheroids. Light microscopy photographs of the spheroids were taken daily and presented at a magnification of 4×; the measured size of the spheroid is indicated in the lower left corner.

The effect of VSV on 3D tumor spheroids was investigated using two different cell lines, T98G and LN229. The spheroids were infected with VSV at a titer of 10^5^ TCID_50_/mL and observed for 5 days. A clear cytopathic effect of VSV was found in T98G spheroids, manifesting as loosening of the spheroid structure and loss of its original shape, as well as the appearance of a small number of growing cells. In contrast, in an uninfected control group, cell proliferation was observed around the attached tumoroids (Figure 8). Additionally, we noticed necrosis of the spheroids due to viral infection. By day 5, lysis of the surviving cells and further loosening and detachment of the spheroids were observed (Figure 7). A similar pattern was also found in LN229 cells. By day 3, the loosening of the spheroids and loss of their original structure were evident, along with a small population of growing cells. These effects increased by day 5, although we did not see 100% cell death in this cell line (Figure 9).

### 2.6. The Effect of Protoporphyrin IX on 3D Spheroids and the Distribution of the Porphyrin over 3D Spheroids

**Figure 10:**
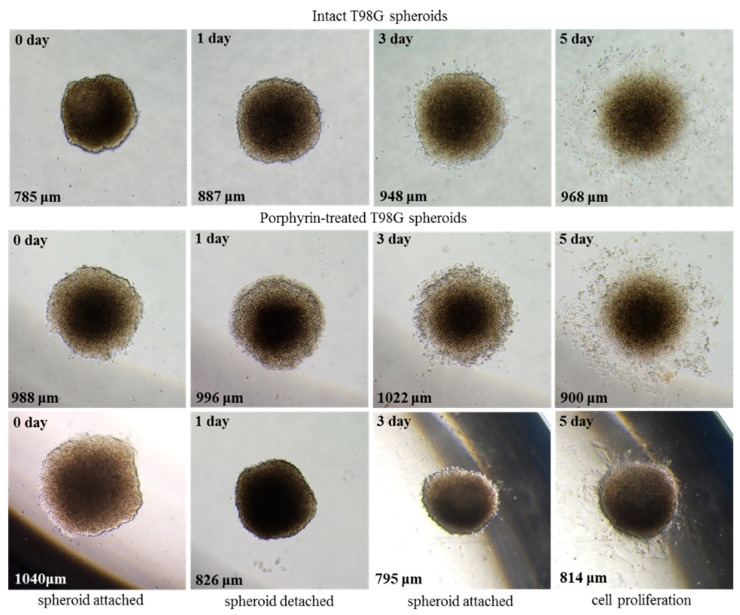
The effect of photodynamic therapy with protoporphyrin IX on T98G spheroids. The spheroids were incubated with protoporphyrin IX at a concentration of 10 μg/mL in PBS (with 1% DMSO). After an hour of incubation, the spheroids were irradiated with light at a wavelength of 450 nm, placed in growth media, and compared with control uninfected spheroids. Light microscopy photographs of the spheroids were taken daily and presented at a magnification of 4×; the measured size of the spheroid is indicated in the lower left corner.

Next, we investigated the effect of protoporphyrin IX as a monotherapy on T98G spheroids. We added protoporphyrin IX to the spheroids at a concentration of 10 μg/mL and incubated them for one hour, followed by irradiation at a wavelength of 450 nm. In some cases, we observed no cytotoxic effect on the attached spheroids, and the cells continued to grow similarly to the control group (Figure 10). However, in other cases, the spheroids detached from the surface of the plate a day after treatment, appeared to die, and then reattached and began to grow slowly (Figure 10).

**Figure 11:**
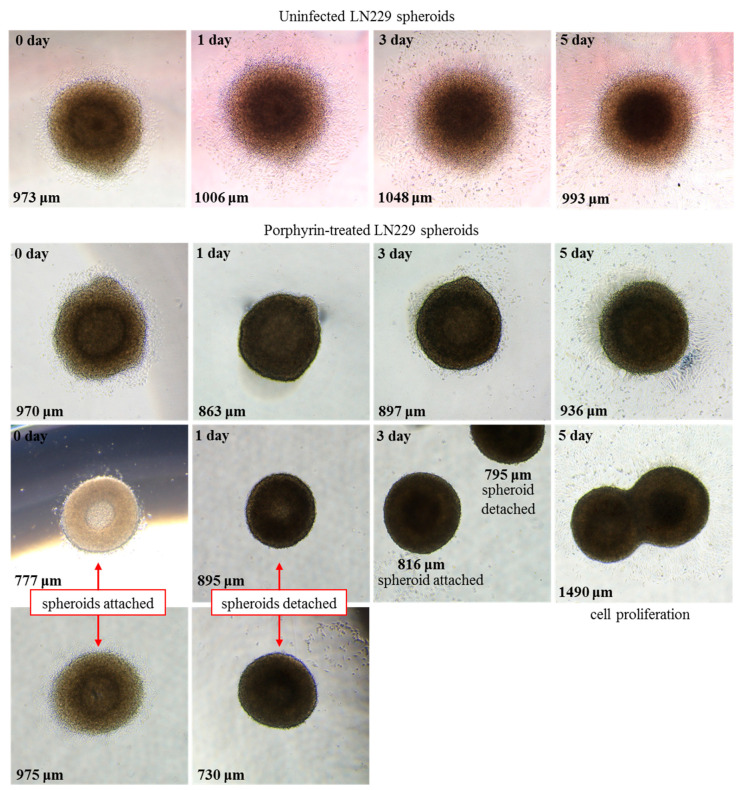
The effect of photodynamic therapy with protoporphyrin IX on LN229 spheroids. The spheroids were incubated with protoporphyrin IX at a concentration of 10 μg/mL in PBS (with 1% DMSO). After an hour of incubation, the spheroids were irradiated with light at a wavelength of 450 nm, placed in growth media, and compared with control uninfected spheroids. Light microscopy photographs of the spheroids were taken daily and presented at a magnification of 4×; the measured size of the spheroid is indicated in the lower left corner.

In contrast to T98G, in the case of LN229 spheroids that did not detach, we observed slower cell proliferation compared to control tumoroids (Figure 11). Additionally, in a well containing two spheroids similar to T98G, we observed detachment, followed by reattachment, fusion, and proliferation of a cell layer. Therefore, photodynamic therapy with protoporphyrin IX as a photosensitizer exerts a cytotoxic effect on tumoroids. However, we did not observe complete cell death. We believe that with increased concentrations, cytotoxic effects would also increase.

### 2.7. The Combination of Protoporphyrin IX and VSV Enhances the Lysis of 3D Spheroids

**Figure 12:**
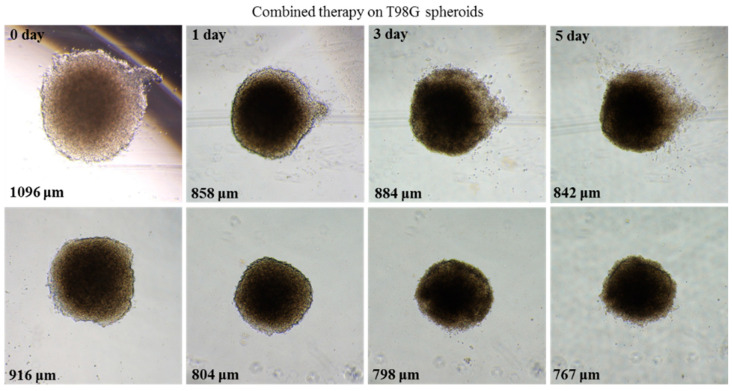
The effect of combination therapy on T98G spheroids. Spheroids were incubated with protoporphyrin IX at a concentration of 10 µg/mL in PBS (with 1% DMSO) for an hour. After incubation, the spheroids were irradiated with light at a wavelength of 450 nm, followed by the addition of VSV at a titer of 10^5^ TCID_50_/mL, and compared with control uninfected spheroids. Light microscopy photographs of the spheroids were taken daily and presented at a magnification of 4×; the measured size of the spheroid is indicated in the lower left corner.

**Figure 13:**
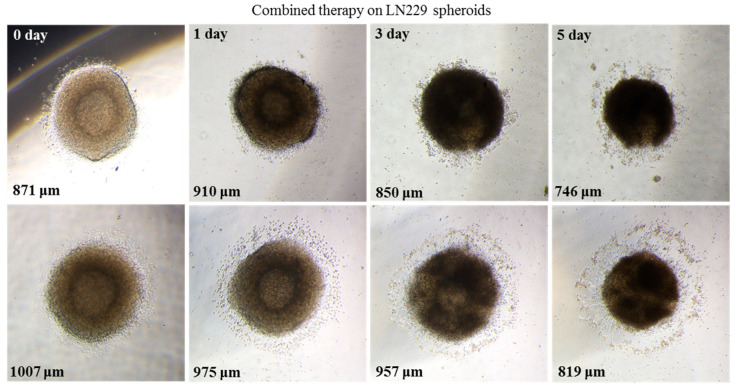
The effect of combination therapy on LN229 spheroids. Spheroids were incubated with protoporphyrin IX at a concentration of 10 µg/mL in PBS (with 1% DMSO) for an hour. After incubation, the spheroids were irradiated with light at a wavelength of 450 nm, followed by the addition of VSV at a titer of 10^5^ TCID_50_/mL, and compared with control uninfected spheroids. Light microscopy photographs of the spheroids were taken daily and presented at a magnification of 4×; the measured size of the spheroid is indicated in the lower left corner.

We investigated the effects of combined therapy with protoporphyrin IX and VSV on T98G and LN229 tumor spheroids. After incubating the spheroids with protoporphyrin for one hour at a concentration of 10 μg/mL, we irradiated them with 450 nm wavelength light and then added the virus at a titer of 10^5^ TCID_50_/mL. Then, we monitored the spheroids for five days. Unlike the monotherapy, we did not see any significant cell proliferation in the spheroids treated with combined therapy (Figure 12 and Figure 13). Some spheroids detached and reattached, similar to what we saw with monotherapy using porphyrin. However, further observation showed only loosening and loss of the native structure of the spheroids, and no proliferation of the cellular layer. By day five, living cells were almost absent and had an abnormal shape.

## 3. Discussion

The oncolytic effects of various variants of the vesicular stomatitis virus (VSV) on tumors of different etiologies are well known [31,36]. Our goal was to combine viral and photodynamic therapy for tumors using the oncolytic potential of VSV along with its vector capabilities. The first task was to preliminarily evaluate if the combination of porphyrin with VSV enhanced tumor toxicity. The separate effects of VSV and porphyrin were studied on cell lines and compared to those of the conjugated form of VSV with porphyrin.

Initially, the oncolytic properties of VSV on human glioblastoma cell lines GL6, LN229, T98G, and human neuroblastoma SH5y5y and Kelly, as well as on the mouse glioma cell line GL261, were tested. The VSV showed an oncolytic effect on all the cell lines, with a strong lytic effect at a virus titer of 10^5^ TCID_50_/mL. VSV titer of 10^4^ TCID_50_/mL was sufficient for neuroblastoma cell death. Interestingly, an increase in virus titer from 10^5^ to 10^7^ TCID_50_/mL was associated with a decrease in the oncolytic activity of VSV on the glioblastoma cells. This may be due to the formation of large numbers of non-infectious virus particles that interfere with the entry of fully formed virions into cells. However, this dependence was not observed for the neuroblastoma cells.

For further investigation of the combined effects of VSV and protoporphyrin IX, we selected two well-characterized human glioblastoma cell lines of different origins: T98G fibroblast and LN229 epithelial cell lines.

The cytotoxicity and phototoxicity of protoporphyrin IX were evaluated on T98G and LN229 cells. The porphyrin exhibited dark toxicity with an IC50 of about 48 ± 7 μg/mL for T98G cultures, while the IC50 for LN229 was 40 ± 6 μg/mL. To assess the phototoxicity of the porphyrin, we selected a concentration range of 0.5 to 16 μg/mL, as other studies have shown that porphyrin derivatives exhibit toxicity at these concentrations [37]. The phototoxicity of protoporphyrin IX was determined with an IC50 of 5 ± 1 μg/mL for T98G and about 3 ± 1 μg/mL for LN229, which is comparably quite high, substantiating the choice of protoporphyrin IX as a photosensitizer for PDT.

In order to facilitate porphyrin delivery to tumors and combine photodynamic and viral therapy, protoporphyrin IX was conjugated to VSV. First, the ability of VSV to interact with small molecules without losing its infectivity was tested. For this model reaction, we chose Cyanine5.5 as a fluorescent dye. The conjugate of VSV with Cyanine5.5 was obtained. The fluorescence spectrum of the VSV–Cyanine5.5 conjugate in PBS buffer fully corresponded to that of the pure Cyanine5.5 dye, with a maximum at 704 nm and a shoulder at 752 nm (Figure 3a). This confirmed the successful incorporation of the dye into the virus.

The fluorescent signal generated by the conjugate made it possible to evaluate its infectivity. Viral particles adsorbed onto the cell surface, and the cytopathic effect of the VSV conjugate was observed, which is characteristic of viral infection. However, conjugation with the cyanine dye resulted in a 5-fold decrease in virus activity. Nevertheless, the ability of VSV to form functional conjugates was confirmed, and this was subsequently realized with protoporphyrin IX.

The conjugation of VSV with protoporphyrin IX proceeded similarly to that of Cya-nine 5.5. The fluorescence spectrum of the VSV–porphyrin conjugate in PBS buffer was similar to that of the pure porphyrin, but it was bathochromically shifted by 14 nm, which may be due to the interaction between the protoporphyrin and the viral environment (Figure 3b). The fluorescence spectrum confirmed the formation of the conjugate of VSV and porphyrin. The conjugate remained infectious, and a viral cytopathic effect was observed on the second day after infection. However, titration of the conjugate showed an 8-fold decrease in virus titer, likely due to the toxic effects of the porphyrin derivative on VSV [38]. The phototoxic properties of the VSV conjugate with protoporphyrin IX was tested on the T98G cell line. Cell lysis was detected upon light irradiation of the infected cells. However, complete cell death was not detected. This could be due to the very low porphyrin concentration provided by the conjugate. The photosensitizer concentration plays a key role in the efficiency of photodynamic therapy; however, an increase in the concentration of the porphyrin demands a proportional increase in virus loading, which is harder to achieve.

Thus, targeted photodynamic monotherapy with VSV proved ineffective due to the limited concentration of the photosensitizer provided by the virus, particularly when employing standard amounts of the virus. Moreover, the results indicated that conjugating porphyrin with the virus resulted in a significant reduction in its activity, rendering it unsuitable for combination therapy using this conjugate. Therefore, the combination therapy involving sequential treatment by porphyrin and virus was studied next.

The combined lytic effect of VSV treatment and photodynamic therapy, using protoporphyrin IX as a photosensitizer, was studied in T98G and LN229 cell lines with the help of the MTT assay. The lytic effect increased with increasing concentrations of porphyrin and virus titer, similar to what was observed with monotherapy. However, we observed a greater lytic effect when both treatments were combined compared to either treatment alone. When the virus was added immediately after protoporphyrin, there was either a slight increase in lytic activity or no increase at all. However, when VSV was added one day after protoporphyrin, it enhanced the lytic effects significantly. This effect could be related to the toxic effect of the intermediates formed during the photodynamic treatment of the virus. Reactive oxygen species (ROS) are formed during irradiation of the photosensitizer in the presence of oxygen [39]. These ROS participate in cell damage and disappear after a day. Therefore, a day’s delay should be used after photodynamic therapy for viral treatment in combination therapy. ROS influence also takes time, after which cancer cells become more susceptible to viral treatment. ROS can directly damage DNA or alter its repair systems. They can also react with proteins, carbohydrates, and lipids, inducing an imbalance in redox homeostasis and altering DNA [40]. Subsequent viral treatment of damaged cells becomes more effective.

**Figure 14:**
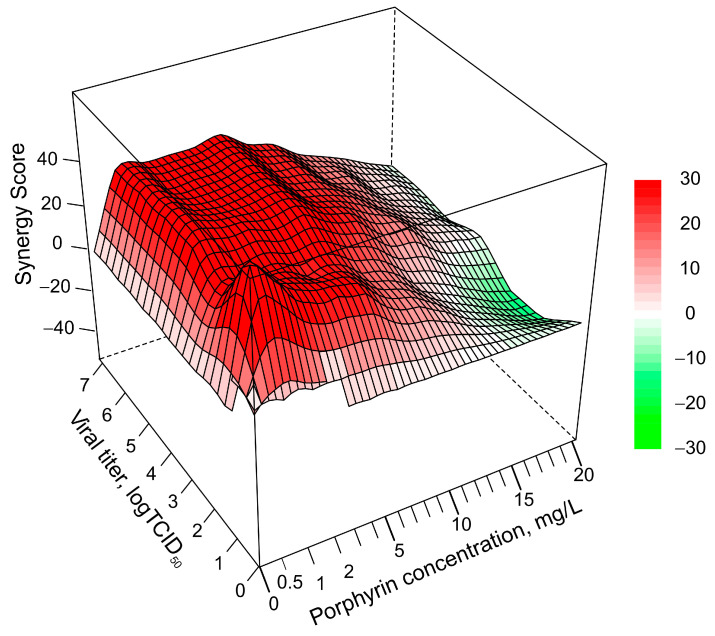
A 3D plot of the synergy score dependence of the concentrations of porphyrin and VSV based on the Loewe additivity model; obtained from the results of the combined VSV and PDT treatment of T98G cell lines.

The synergistic effect of the PDT and viral treatment of T98G cells was analyzed using the Loewe additivity model. The calculation was performed using the SynergyFinder R package version 3.14.0 [41]. VSV treatment a day after PDT was taken into account. The synergy score depended on the concentration of the agents, as shown in the 3D plot (Figure 14).

The highest synergy score was observed at those concentrations where the viability dependence on concentration was highest. The mean synergy score was 20, indicating a significant synergistic effect of the combined treatment of the T98G cell line. Another cell line, LN229, showed an abnormal dependence of viability on viral titer during combined therapy. A VSV titer of 10^3^ TCID_50_/mL had a stronger effect than a titer of 10^4^ TCID_50_/mL, which interfered with the expected behavior and hindered synergy analysis.

Multicellular tumor spheroids often exhibit characteristics similar to those of solid tumors in vivo, making them a more suitable model for studying VSV treatment and photodynamic therapy than conventional cell cultures. Therefore, studies were also performed on spheroids of T98G and LN229 cells using VSV and photodynamic therapy. A clear cytotoxic effect of VSV was observed on both cell lines when monitoring cell proliferation using optical microscopy. VSV addition led to the necrosis of T98G tumoroids 5 days after viral treatment. However, complete cell death was not achieved after 5 days of VSV treatment of LN229 tumoroids. Therefore, there is a need for additional treatment, such as photodynamic therapy, to achieve complete tumor cell death.

Photodynamic therapy with protoporphyrin IX as a photosensitizer also has a cytotoxic effect on both T98G and LN229 tumoroids, but it seems to not be as effective as viral treatment. In contrast to the viral treatment, T98G spheroids survived better compared to LN229. However, complete cell death was not achieved for either cell line. We believe that increasing the porphyrin concentration may enhance the cytotoxic effects and lead to complete cell death. Overall, photodynamic therapy as a monotherapy and monotherapy with VSV were shown to be insufficient for tumoroid treatment. Therefore, a combined therapy approach is necessary. We investigated the effects of this combined treatment.

The combination therapy, using photodynamic therapy with protoporphyrin IX as a photosensitizer in the first stage and VSV treatment in the second stage, was performed on T98G and LN229 tumor spheroids. In contrast to either type of monotherapy, the combination therapy led to complete cell death of tumoroids from both cell lines after 5 days. Therefore, combination therapy significantly enhances the lytic effects of viral and photodynamic treatments on tumor spheroids.

Thus, it was found that VSV had a clear viral cytopathic effect on T98G and LN229 cell lines, including their 3D spheroids. Photodynamic therapy with protoporphyrin IX as a photosensitizer also had a clear cytotoxic effect on these cells and, to a lesser extent, on the spheroids. However, only the combination therapy involving sequential photodynamic and viral treatment achieved the required result of complete cell death. The combination therapy was shown to significantly improve efficacy.

Photodynamic therapy is a promising treatment for various cancers, including brain tumors. However, like other therapies, glioblastomas have developed resistance mechanisms that prevent their complete elimination [42]. Glioblastoma stem cells, which are associated with tumor regrowth, have shown resistance to most treatments developed for glioblastomas, including PDT. The effectiveness of a treatment approach depends on the ability to target these cells [43]. Therefore, the lack of clear effectiveness of PDT in improving overall survival has limited its widespread adoption and implementation as a standard treatment for glioblastomas [22]. The effectiveness of PDT could be increased by combining it with other forms of therapy [44]. The results of the current study clearly demonstrated that the combination of PDT with oncolytic virus-based treatment shows a significant synergy, leading to improved efficiency.

## 4. Materials and Methods

### 4.1. Cell Lines, Culture, and Conditions

The Vero green monkey kidney epithelial cell line (obtained from the American Type Culture Collection—ATCC, CCL-81) was used for the propagation and titration of vesicular stomatitis virus (VSV) using cytopathic effect. The GL-6 human glioblastoma cell line (Chumakov FSC R&D IBP RAS (Institute of Poliomyelitis)), T98G, LN229 glioblastoma lines (ATCC), mouse glioma cell line GL261, KELLY and SH-SY5Y human neuroblastoma cell lines (Chumakov FSC R&D IBP RAS (Institute of Poliomyelitis)) were used to assess sensitivity to VSV. These cell lines were also used to study the lytic effects of photodynamic therapy with porphyrins and the efficacy of combination therapies. The cells were grown in a nutrient medium, DMEM (Chumakov FSC R&D IBP RAS (Institute of Poliomyelitis)), supplemented with 2 mM/L L-glutamine (#F032, PanEco, Moscow, Russia), penicillin (250 IU/mL) (#A065p, PanEco, Moscow, Russia), streptomycin (200 μg/mL) (#A065p, PanEco, Moscow, Russia), and 5% fetal bovine serum (FBS, #24120-72, Gibco, Grand Island, NY, USA). The culture was maintained at optimal growth conditions of 37 °C and 5% CO_2_. As a supportive medium for cells infected with viruses, the “IGLA MEM” medium (Chumakov FSC R&D IBP RAS (Institute of Poliomyelitis)) was used. This VSV culture medium is identical to DMEM in composition.

### 4.2. Viral Strains

Vesicular stomatitis virus (Indiana strain) (VSV), obtained from the collection of the Chumakov Federal Scientific Center for Research and Development of Immune-and-Biological Products, was cultured on Vero cells in the “IGLA MEM” medium (Chumakov FSC R&D IBP RAS (Institute of Poliomyelitis)) supplemented with 2 mM/L L-glutamine (#F032, PanEco, Moscow, Russia), penicillin (250 IU/mL) (#A065p, PanEco, Moscow, Russia), streptomycin (200 μg/mL) (#A065p, PanEco, Moscow, Russia), and 5% fetal bovine serum (FBS, #24120-72, Gibco, NY, USA). The culture was maintained at optimal growth conditions of 37 °C and 5% CO_2_. The titer of the virus and its conjugates was determined using the Reed–Muench method.

### 4.3. Sensitivity of Cell Lines to Vesicular Stomatitis Virus

96-well plates containing cells from transplanted tumor lines were infected with 10-fold serial dilutions of VSV in six repeats. The virus was allowed to adsorb for 1 h, after which the virus-containing fluid was removed and the plates were washed with “IGLA MEM” medium. The cells were then cultured in VSV culture medium for 3 days. After this period, cell viability was assessed using the MTT assay, using the MT reagent (#O104, PanEco, Moscow, Russia). A solution was prepared using phosphate buffer at a working concentration of 5 mg/mL. The optical density was measured at a wavelength of 595/650 nm using the Bio-Rad iMark tablet reader (Bio-Rad Laboratories, Inc., Hercules, CA, USA). The results were compared to the average optical density of the control, which was taken as 100%.

### 4.4. Sensitivity of Cell Lines to Protoporphyrin IX

Protoporphyrin IX was dissolved in DMSO at an initial concentration of 1 g/L, and further dilutions of protoporphyrin IX were prepared in phosphate-buffered saline (PBS) containing no more than 10% DMSO. Solutions of protoporphyrin IX at different concentrations were then added to 96-well plates containing cells from transplanted tumor lines. PBS with 10% DMSO was used as a control series. After one hour of incubation, the cells were either irradiated with light at a wavelength of 450 nm (for phototoxicity determination) or kept in the dark (for dark toxicity determination). Protoporphyrin IX and PBS were then removed. The cell lines treated with protoporphyrin IX were then cultured in growth medium for one day, after which their viability was determined using the MTT assay, which is similar to the method used for determining the sensitivity of the cell lines to vesicular stomatitis virus (VSV). The experiments were repeated six times at each porphyrin concentration.

### 4.5. Combination VSV and Photodynamic Treatment of Cell Lines

Dilutions of protoporphyrin IX and VSV were prepared similarly. Solutions of protoporphyrin IX were added to 96-well plates containing cells of transplanted tumor lines at different concentrations. PBS with 10% DMSO was used as the cell and virus controls, and protoporphyrin IX at a concentration of 2 μg/mL was used as a porphyrin control group. After an hour of incubation, the cells were irradiated with 450 nm light. Protoporphyrin and PBS were then removed.

The cell lines were then infected with VSV at titers of 10^3^ and 10^4^ TCID_50_/mL immediately after removing protoporphyrin or after incubating the cells in growth medium for a day. The virus was adsorbed for one hour, and the virus-containing medium was removed. The cells were washed with “IGLA MEM” medium and cultured in VSV culture medium. The virus was not added to the control groups. After three days, cell viability was determined using the MTT assay, following the same procedure as in the previous experiments. The experiments were repeated six times.

### 4.6. Procedures for the Synthesis of Conjugates of VSV with Dyes

#### 4.6.1. Clarification and Concentration of VSV

The virus-containing liquid obtained after cultivation was centrifuged at 3500 rpm for 1 h at 4 °C. The resulting supernatant was then concentrated by ultracentrifugation using the SW28 rotor at 25,000 rpm for 2.5 h at 4 °C. The concentrated virus was dissolved in phosphate-buffered saline (PBS) with pH 8.2 and then ultracentrifuged again on the SW55 rotor at 35,000 rpm for 2.5 h at 4 °C. Afterwards, the concentrated VSV was dissolved in PBS and the resulting concentrated virus was titrated using the Reed–Mench method.

#### 4.6.2. VSV Labeling with Cyanine5.5 Fluorescent Label

Cyanine5.5 NHS-ether (Lumiprobe) fluorescent label (**1**) (0.4 mg, 0.0055 mmol) was dissolved in 1 mL of absolute DMSO. Then, 50 μL of the resulting solution was added to 500 μL of VSV (4 × 10^9^ TCID_50_/mL) and stirred at room temperature for 3 h. Then, the mixture was purified by ultracentrifugation on the SW55 rotor at 35,000 rpm for 2.5 h at 4 °C, followed by dissolving the residue in PBS and concentrating the conjugate on a centrifuge concentrator with a pore size of 0.45 μm at 7000 rpm for 15 min. As a result, the conjugate of VSV with Cyanine5.5 (**2**) was obtained.

#### 4.6.3. Synthesis of Protoporphyrin IX (**4**) [45]

In a round bottom flask, 153 mg (0.235 mmol) of hemin (**3**) was dissolved in 6 mL of formic acid. To this solution, 157 mg (2.82 mmol) of iron powder was added in two portions within 5 min with stirring at 60 °C. After boiling for 30 min, the reaction mixture was cooled to room temperature, then filtration was carried out and hot formic acid was used for washing. A solution of 53 g of sodium acetate in 60 mL of distilled water was then added to a mother liquor and the resulting mixture was kept in the fridge for 24 h to afford precipitation of the product. The product was collected through filtration and washed with water before being dried. Protoporphyrin IX (**4**) was obtained with a yield of 131 mg (99%). ^1^H NMR (600 MHz, TFA-d1, δ): 3.45 (2t, 2 CH_2_); 3.82 (s, CH_3_); 3.85 (s, CH_3_); 3.88 (s, CH_3_); 3.91 (s, CH_3_); 4.73 (2t, 2 CH_2_); 6.43–6.70 (m, 4H, 2 CH_2_=C); 8.28–8.40 (m, 2H, 2 CH=C); 11.08, 11.11, 11.15, and 11.27 (4 s, 4 CH). MALDI-MS (*m*/*z*): Calculated: [M]+ = 562.41; Observed: [M]+ = 562.57.

#### 4.6.4. Synthesis of Protoporphyrin IX N-Succinimide Ester (**5**) [46]

In a Schlenk flask, 42 mg (0.075 mmol) of protoporphyrin IX (**4**) was dissolved in 7 mL of CH_2_Cl_2_ and 2 mL of DMSO. To this solution, 23 mg (0.187 mmol) of 4-dimethylaminopyridine (DMAP) was added along with a solution of 70 mg (0.448 mmol) of 1-ethyl-3-(3-dimethylaminopropyl)carbodiimide (EDC) hydrochloride in 2 mL of DMSO. The flask was then placed in an ice bath and stirred for 10 min. Then, 52 mg (0.448 mmol) of N-hydroxysuccinimide (NHS) was added to the flask and kept in an ice bath for 3 h with stirring. After this, the ice bath was removed and the solution was stirred for another 48 h at room temperature. A 10 mL volume of diluted ethanolic solution in water (EtOH/H_2_O = 75:25% *v*/*v*) was added to the flask to afford precipitation of the product. The product was collected through filtration and washed with ethanol before being dried. The protoporphyrin IX N-Succinimide Ester (**5**) was obtained with a yield of 39 mg (69%). MALDI-MS (*m*/*z*): Calculated: [M]+ = 756.30; Observed: [M]+ = 756.31. UV-Vis (DMF, λ, nm): 404, 623, 576, 542, 506. Molar extinction coefficient (λ = 404 nm; DMF; molL^−1^ cm^−1^): 195,800.

#### 4.6.5. Conjugation of Protoporphyrin IX N-Succinimide Ester with VSV

Protoporphyrin IX N-Succinimide ester (**5**) solution (300 μL), at a concentration of 10 μg/mL, and 1.2 mL of PBS with a pH of 8.3 were added to 500 μL of VSV (10^8^ TCID_50_/mL), left stirring at room temperature for 3 h, and then cooled to 4 °C and concentrated in the same manner as the VSV conjugate with Cyanine5.5, yielding a conjugate of protoporphyrin IX with VSV (**6**).

### 4.7. Fluorescence Microscopy

The Vero cell line with a confluence of 30–50% was infected with the obtained conjugates. After an hour of incubation, the cells were washed three times with PBS cell culture and then the FluoroBrite™ DMEM medium (Gibco) was added. The adsorption of conjugates on the cell surface was evaluated using fluorescence microscopy on the Celena X imaging system (Logos Biosystems, Anyang-si, Gyeonggi-do, Republic of Korea) on the Cy5 channel (650–690 nm).

### 4.8. Determination of the Sensitivity of T98G Culture to the Resulting Conjugate of Activated Protoporphyrin IX Ester with VSV

The T98G monolayer was infected with the resulting VSV–protoporphyrin IX conjugate and incubated for an hour. This was followed by washing the PBS cell culture 3 times and adding a VSV culture medium. Then, the cells were irradiated with light at a wavelength of 450 nm and incubated for a day. The lytic effect of the conjugate was evaluated using an inverted OLYMPUS CKX53 biological microscope (Olympus, Tokyo, Japan).

### 4.9. Determination of the Sensitivity of Tissue Spheroids

#### 4.9.1. Formation of Tissue Spheroids

Tissue spheroids were formed using agarose molds created using silicone matrices (courtesy of I.V. Zubarev), according to the instructions provided by the manufacturer (Laboratory for Biotechnological Research 3D Bioprinting Solutions, Moscow, Russia) [47].

#### 4.9.2. Sensitivity to Vesicular Stomatitis Virus

Spheroids were placed in 24-well plates, with 1–2 spheroids per well, and infected with VSV at a concentration of 10^5^ TCID_50_/mL, in six repeats, with the addition of VSV culture medium. A visual assessment of the cytopathic effect of the virus on the spheroids was then performed for five days.

#### 4.9.3. Sensitivity to Photodynamic Treatment

Protoporphyrin IX at a concentration of 10 μg/mL in PBS (with 1% DMSO) was used to treat spheroids in a 24-well plate, with 1–2 spheroids per well and 6 replicates. PBS (1% DMSO) served as a control for the spheroid group. After an hour of incubation, the spheroids were irradiated at a wavelength of 450 nm. Protoporphyrin IX and PBS were then removed, and a growth medium was added. A visual assessment of the effect of protoporphyrin IX on the spheroids was conducted for 5 days.

#### 4.9.4. Combination VSV and Photodynamic Treatment

Protoporphyrin IX at 10 µg/mL in PBS (1% DMSO) was applied to spheroids in a 24-well plate at a density of 1–2 spheroids/well in six replicates. After an incubation period of one hour, the spheroids were subjected to light irradiation at a wavelength of 450 nanometers. Protoporphyrin IX was then removed, followed by the addition of VSV (10^5^ TCID_50_/mL). A VSV culture medium was used for incubation for 5 days, during which time a visual assessment of the lysis effect was performed.

### 4.10. Microscopy

Visual inspection and photography of cells and spheroids were performed using an inverted OLYMPUS CKX53 biological microscope equipped with a digital camera and ADFImageCapture software version 4.11.21522.20221011.

### 4.11. Statistical Analysis

Statistical evaluation of the data was carried out in the GraphPad Prism 8 program using ANOVA analysis of variance. Synergy analysis was performed using the SynergyFinder R package [41].

### 4.12. Fluorescence Spectra

Photoluminescence spectra were recorded on the Fluorolog-3 spectrofluorometer (HORIBA Jobin Yvon S.A.S., Palaiseau, France). The excitation source was a 450 W xenon lamp with Czerny–Turner double monochromators; an R928 photomultiplier and an InGaAs near-IR detector were used to detect the signals.

## 5. Conclusions

This study demonstrated the potential of using vesicular stomatitis virus (VSV) in the treatment of glioblastomas and neuroblastomas through viral therapy. The VSV exhibited an oncolytic effect on glioblastoma cell lines T98G and LN229 when the virus titer was 10^5^ TCID_50_/mL. A VSV titer of 10^4^ TCID_50_/mL proved sufficient for inducing the death of neuroblastoma cells. One of the objectives of this investigation was to explore the feasibility of employing vesicular stomatitis virus (VSV) as a tool for the targeted delivery of protoporphyrin IX, a photosensitizer, to cancerous tumors such as glioblastomas, followed by implementing a combined approach involving viral and photodynamic therapies.

The results indicated that conjugating porphyrin with the virus resulted in a significant reduction in its activity, rendering it unsuitable for combination therapy using this conjugate. In addition, targeted photodynamic monotherapy proved ineffective due to the limited concentration of the photosensitizer provided by the virus, particularly when employing standard amounts of the virus. The concentration of protoporphyrin IX required for efficient photodynamic therapy was found to be greater than 10 μg/mL. However, this concentration can be reduced by an order of magnitude when combined with viral therapy. Therefore, the implementation of combination therapy involving the sequential administration of porphyrin and virus proved to be more efficient. In this approach, medications are administered in a sequential manner: first, porphyrin is administered, followed by irradiation; then, a virus is employed to target tumor cells that have been weakened by the photodynamic therapy. In this instance, there is a marked enhancement in efficacy based on the synergistic effect, which allows the use of a lower viral titer (10^3^–10^4^ TCID_50_/mL) and a lower concentration of porphyrin (0.5 μg/mL) to achieve the desired cytotoxic effect. As a result of the combination therapy, fewer side effects are expected from the treatment, which could lead to a more effective approach for treating highly aggressive tumors like glioblastomas. The limitations of PDT, which are associated with the development of glioblastoma resistance, can be overcome through the synergistic effects of combined viral and photodynamic therapy. Therefore, this combination therapy holds great promise for future treatment of these tumors.

## Data Availability

Data supporting this study are included within the article.
